# High Protein- and High Lipid-Producing Microalgae from Northern Australia as Potential Feedstock for Animal Feed and Biodiesel

**DOI:** 10.3389/fbioe.2015.00053

**Published:** 2015-05-18

**Authors:** Van Thang Duong, Faruq Ahmed, Skye R. Thomas-Hall, Simon Quigley, Ekaterina Nowak, Peer M. Schenk

**Affiliations:** ^1^Algae Biotechnology Laboratory, School of Agriculture and Food Sciences, The University of Queensland, Brisbane, QLD, Australia; ^2^School of Agriculture and Food Sciences, The University of Queensland, Brisbane, QLD, Australia

**Keywords:** 18S rDNA sequencing, animal feed, biodiesel, fatty acid methyl esters, microalgae, protein-rich biomass, triacylglyceride

## Abstract

Microalgal biomass can be used for biodiesel, feed, and food production. Collection and identification of local microalgal strains in the Northern Territory, Australia was conducted to identify strains with high protein and lipid contents as potential feedstock for animal feed and biodiesel production, respectively. A total of 36 strains were isolated from 13 samples collected from a variety of freshwater locations, such as dams, ponds, and streams and subsequently classified by 18S rDNA sequencing. All of the strains were green microalgae and predominantly belong to *Chlorella* sp., *Scenedesmus* sp., *Desmodesmus* sp., *Chlamydomonas* sp., *Pseudomuriella* sp., *Tetraedron caudatum*, *Graesiella emersonii*, and *Mychonastes timauensis*. Among the fastest growing strains, *Scenedesmus* sp. NT1d possessed the highest content of protein; reaching up to 33% of its dry weight. In terms of lipid production, *Chlorella* sp. NT8a and *Scenedesmus dimorphus* NT8e produced the highest triglyceride contents of 116.9 and 99.13 μg mL^−1^ culture, respectively, as measured by gas chromatography–mass spectroscopy of fatty acid methyl esters. These strains may present suitable candidates for biodiesel production after further optimization of culturing conditions, while their protein-rich biomass could be used for animal feed.

## Introduction

Microalgae occur widely in a variety of natural and man-made environments, including fresh, brackish, sea and waste water, as well as in soil and on other organisms. Most microalgae can be found in freshwater and marine habitats such as lakes, rivers, streams, pond, estuary, and coastal areas. Richmond (2004) reported that there could be about 50,000 species, although at present about 30,000 species have been identified and analyzed.

Based on the large amount of research, the potential of microalgae’s application to the biofuel industry has increased in recent years. There are many microalgae that can accumulate large amounts of lipids in their cells (Sheehan et al., 1998; Lim et al., 2012). The lipid content depends on the specific algal strain and their growth conditions, with average contents ranging from 2 to 75% of dry weight (DW) under exceptional circumstances, but are typically between 10 and 30% DW (Chisti, 2007; Li et al., 2008; Schenk et al., 2008; Sharma et al., 2012). For instance, the green alga *Botryococcus braunii* can produce hydrocarbons up to 75% of their DW. This species is being considered as a possible source for future biodiesel production (Chisti, 2007), but its growth rates are considered not competitive compared to many other microalgae.

Freshwater strains can produce high amounts of protein and other bio-products that have valuable properties in industry, such as antioxidants (e.g., carotenoids) and emulsifiers that are used for the alimentary industry (Chisti, 2007; Ahmed et al., 2014), omega 3 and omega 6 fatty acids as nutraceuticals (Adarme-Vega et al., 2014) or lipid for biodiesel feedstock (Chisti, 2007; Sharma et al., 2014). Using local strains has been demonstrated to ensure dominance and high adaptability to local environmental and climatic conditions and should be the preferred option to prevent the invasion of non-indigenous species in the environment. Advantages of microalgae compared to first generation biofuel crops, include their high areal productivity resulting in less land use than other crops, a wide range of adaptation in different environments including their ability for rapid growth in brackish, saline, or waste water without the need to compete for arable land or biodiverse landscapes (Rodolfi et al., 2009; Mata et al., 2010).

The application of microalgae in tropical aquaculture is rapidly increasing. Understanding their chemical composition including protein and fatty acids profiles enables effective screening of candidate algal strains with the aim to optimize conditions for large-scale cultivation, including suitable methods for lipid induction and extraction (Renaud et al., 1994; Sharma et al., 2012; Ghasemi Naghdi et al., 2014). Australia also has a high demand for protein-rich feed for livestock, in particular, for cattle in the Northern Territory where no local protein-rich feeding crops are available during the dry season. An alternative source of protein-rich feed can potentially be provided from on-farm microalgae cultivation.

Molecular DNA-based techniques are becoming increasingly popular in phycological classification studies (Tang et al., 2011). Comparisons of morphological similarities between microalgal species have been used frequently but the results sometimes generate mistakes in taxonomy because of the apparent morphological similarities to other organisms (Hu et al., 2008). Along with morphological techniques, genetic profiling of microalgae is a great tool for their classification (González López et al., 2010).

In a future scenario, it might be possible to use microalgae as feedstock for oil extraction for biodiesel, while the remaining biomass can be used as animal feed. The purpose of this study was to collect, identify, and characterize local microalgal strains from outback Australia that have the ability to accumulate high amounts of valuable products such as protein for feed production and fatty acids for biofuel industry. Protein and fatty acids methyl esters profiles were also determined and evaluated for their suitability as biodiesel feedstock.

## Materials and Methods

A total of 13 samples were collected in October 2012 from the surface and bottom ground of freshwater dams, streams, and ponds in the Northern Territory, Australia. Sample locations included Brunchilly out-station (S Kidman and Co.), Tennant Creek, NT (GPS 18°52′03″S, 134°30′22″E; sampled from bottom of “Turkey nest”; strain names: NT1x), Katherine Research Station (South Stuart Highway, Katherine; GPS 14°28′21″S, 132°18′17″E; sampled from bottom of “Cooler”; strain names: NT3x); Kidman Springs (Buchanan Highway; GPS 16°07′04″S 130°57′30″E; sampled from surface and subsurface of “Suppleject Dam”; strain names NT5x and NT6x, respectively) and Douglas Daly Research Farm (Jungwa Road, Douglas Daly PMB 105, Winellie; GPS 13°49′59″S 131°11′12″E; sampled from bottom of “Gamba dam”; strain names: NT8x). Samples were preserved in the dark until transferred to the laboratory for analyses.

### Isolation of pure microalgal strains

Single cells were isolated by micropipette on a micromanipulator with an inverted microscope and grown on 96 well-plates before transferred to 100 mL flasks in bold’s basal medium (BBM) for cultivation of pure clonal algal cultures at 25°C, 12:12 h light:dark cycle under fluorescent white light (120 μmol photons m^−2^s^−1^), as described previously (Duong et al., 2012; Lim et al., 2012; Salama et al., 2013).

### Classification by DNA sequencing

DNA extraction was conducted at the late exponential phase of cultivation. The cell density at that point was typically 2 × 10^7^ cells mL^−1^. Microalgal cells were extracted by using an DNeasy Plant Kit (Qiagen) following the manufacturer’s instructions. After extraction, genomic DNA within the 18S rRNA region was amplified on a PCR machine by using the following primers: Forward 5′-GCGGTAATTCCAGCTCCAATAGC-3′ and Reverse 5′-GACCATACTCCCCCCGGAACC-3′. The PCR cycling conditions comprised 94°C for 5 min for initialization, 94°C for 30 s for denaturation, annealing at 55°C for 30 s, and 72°C for 1 min for elongation. The final elongation step was at 72°C for 10 min. PCR templates were then purified by using a Wizard SV Gel PCR Clean-Up System (Promega). For sequencing preparation, 5 μL of a 25 ng μL^−1^ PCR product were combined with 1 μL of a 10 μM solution of each of the above primers. The reaction was topped up to 12 μL with Millipore water in a 1.5 mL tube and sent to the Australian Genome Research Facility (AGRF) at The University of Queensland for sequencing. The DNA sequencing data were then analyzed by MEGA 5.2 and the results were compared by BLAST searches with Genbank entries for classification. All of the strains were registered and deposited in Genbank with accession numbers (as shown in the Section “Results”). For sequences with >99% identity match, the species name was adopted, otherwise the genus name to the closest match was used. The maximum parsimony tree was obtained using the Subtree-Pruning-Regrafting algorithm with search level 1 in which the initial trees were obtained by the random addition of sequences (10 replicates). The tree was drawn to scale with branch lengths calculated using the average pathway method and are in the units of the number of changes over the whole sequence. The analysis involved 11 nucleotide sequences. There were a total of 463 positions in the final dataset. Evolutionary analyses were conducted in MEGA5 (Tamura et al., 2011).

### Standard protocol for growth experiments

After obtaining pure cultures, all isolated strains were grown on BBM medium following a standardized cultivation protocol that used bubbling for aeration and mixing. The standard protocol can be briefly described as follows: all strains were inoculated from a recently grown saturated culture and cultured for 3–4 days to reach the end of the exponential growth phase before starting the standard growth experiment. This culture was used as inoculum at a ratio of 1/10 in volume in 400 mL bottles. The bottles were connected to a bubbling system. Cell density was determined daily by using a hemocytometer. Nitrate concentrations were also monitored daily until the nutrient levels reached 0 by using a colorimetric assay (API test kit; Aquarium Parmaceuticals) and a spectrophotometer following the manufacturer’s instructions.

Growth rates were calculated by the following equation (Levasseur et al., 1993)

$$K^{\prime }=\frac{\ln\frac{N2}{N1}}{t2-t1}$$

where *N*1 and *N*2 = cell counts at time 1 (*t*1) and time 2 (*t*2), respectively. Divisions per day can also be calculated once the specific growth rate is known.

$$\text{Division per day}=\frac{K^{\prime }}{\ln2}$$

### FAME analysis

Samples for fatty acid methyl ester (FAME) analyses were collected when lipid accumulation reached its peak, normally after 3–4 days of nutrient starvation. A total of 4 mL of microalgal culture was collected and centrifuged at 8,000 × *g* for 5 min. Biomass was collected and freeze-dried for 30 min. Lipids in the microalgal pellet were hydrolyzed and methyl-esterified in 300 μL of a 2% H_2_SO_4_ in methanol solution for 2 h at 80°C. Prior to the reaction, 50 μg of heneicosanoic acid provided by Sigma, USA was added as internal standard. After the esterification step, 300 μL of 0.9% (w/v) NaCl solution and 300 μL of hexane were added and mixed for 20 s. To separate the phase, samples were then centrifuged at 16,000 × *g* for 3 min. A total of 1 μL of the hexane layer was injected into an Agilent 6890 gas chromatograph coupled to a 5975 MSD mass spectrometer. The running conditions were followed using Agilent’s RTL DBWax method as described previously (Lim et al., 2012).

### Protein analysis

Protein contents in the algal biomass were measured following the protocol described by González López et al. (2010) with modifications. In brief, freeze-dried biomass (10 mg) was milled and protein was extracted by incubation in 10 mL lysis buffer (containing 5 mL L^−1^ of Triton X-100, 0.3722 g L^−1^ of ethylenediaminetetraacetic acid disodium salt, 0.0348 g L^−1^ of phenyl methyl sulfonyl fluoride) for 20 min. A 0.1 mL portion of this solution was placed in a 1.5 mL Eppendorf tube and 0.1 mL SDS solution was added and the mixture was vortexed. The mixture was then used for measurement of protein concentration following the protocol described in the CB-X Protein Assay Kit (G Biosciences).

The spectrophotometric absorbance was converted to protein concentrations using a calibration curve established with a bovine serum albumin (BSA) standard (2 mg mL^−1^). The protein content of the biomass was calculated using the following equation:

$$\text{Protein}\left(\%\right)=\left(\frac{\mathrm{CVD}}{m}\right)\times 100$$

where *C* = protein concentration (mg L^−1^) obtained from the calibration curve, *V* = volume (L) of the lysis buffer used to resuspend the biomass, *D* = dilution factor, *m* = biomass (mg).

## Results

### *Desmodesmus* sp. and *Scenedesmus* sp. are abundant microalgae in the northern territory, australia

**Figure 1:**
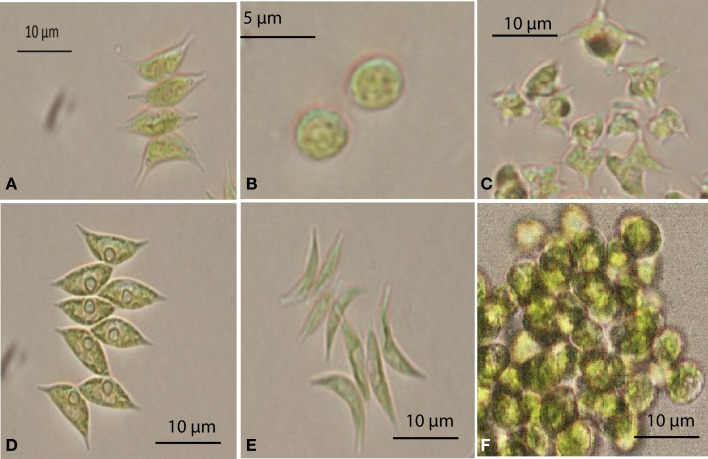
**Selected microalgae isolated from the Northern Territory, Australia, observed under a microscope**. **(A)** *Scenedesmus* sp. NT1d, **(B)** *Chlorella* sp. NT8a, **(C)** *Tetraedron caudatum* NT5, **(D)** *Scenedesmus dimorphus* NT8c, **(E)** *Scenedesmus dimorphus* NT8e, **(F)** *Graesiella emersonii* NT1e.

**Figure 2:**
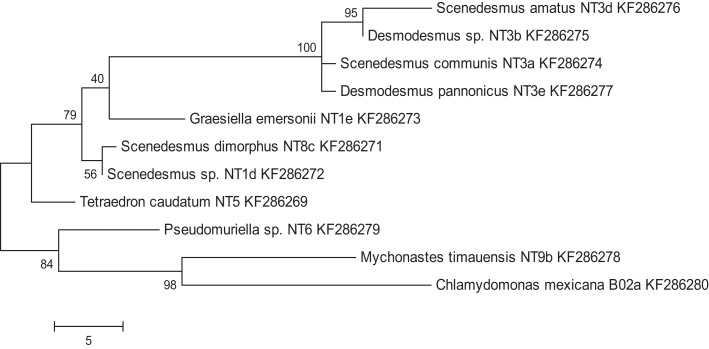
**Maximum parsimony analysis of taxa of isolated microalgae**. Shown are genus, species (if applicable), and strain names, as well as Genbank entry accession numbers. The most parsimonious tree with a length of 96 is shown. The consistency index was 0.761905, the retention index was 0.83871, and the composite index was 0.707661 and 0.639017 for all sites and parsimony-informative sites, respectively. The percentage of replicate trees in which the associated taxa clustered together in the bootstrap test (200 replicates) is shown next to the branches.

A total of 36 algal strains were isolated from 13 samples that were collected at different cattle stations in the Northern Territory, Australia. All strains were maintained in pure conditions for DNA extractions. The results of DNA sequencing show the classification of the strains. All of the isolated strains belonged to the *Chlorophyceae* and *Trebouxiophyceae*. Among these strains, *Desmodesmus* sp., *Scenedesmus dimorphus*, and *Scenedesmus communis* were the most abundant species in the samples. The wide distribution of the strains reflects the wide adaptation ability in the tropical fresh waters of the Northern Territory. The morphology of six strains that displayed rapid growth is illustrated in Figure 1. Following 18S rRNA gene sequencing, the evolutionary relationship of 11 genetically distinct microalgal strains was inferred using the maximum parsimony method (Figure 2).

### Selection of strains with rapid growth rates

**Figure 3:**
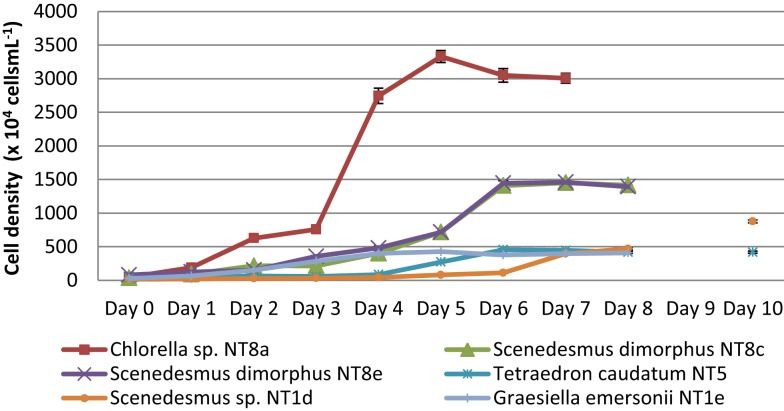
**Growth curves of isolated microalgal strains from the Northern Territory**. Shown are mean values and SEs from three separately grown cultures each.

Microalgae from the Northern Territory that grew well in BBM medium were further characterized and six different strains were chosen for inclusion in the standard protocol (Figure 3). The results from Table 1 show that the specific growth rate of *Chlorella* sp. NT8a was highest, reaching 0.59 day^−1^, followed by *S. dimorphus* NT8c and *S. dimorphus* NT1d at 0.52 and 0.48 day^−1^, respectively. The lowest growth rate among these strain belong to *Tetraedron caudatum* NT5 and *Graesiella emersonii* NT1e at 0.37 and 0.38 day^−1^, respectively. The highest growth rate of *Chlorella* sp. NT8a was also shown in the data by divisions per day and productivity (Table 1).

**Table 1:** **Growth values of isolated microalgae from the Northern Territory selected for further experimentation**.

Strain	Growth rate (μ day^−1^)	Divisions per day	Maximum cell density (×10^6^ cells mL^−1^)	Biomass productivity (g L^−1^ day^−1^)
*Chlorella* sp. NT8a	0.59	0.85	30.50	0.33
*Scenedesmus dimorphus* NT8c	0.52	0.75	14.53	0.07
*Scenedesmus dimorphus* NT8e	0.41	0.59	14.59	0.09
*Tetraedron caudatum* NT5	0.37	0.53	4.57	0.02
*Scenedesmus* sp. NT1d	0.48	0.69	4.69	0.03
*Graesiella emersonii* NT1e	0.38	0.55	4.27	0.14

**Figure 4:**
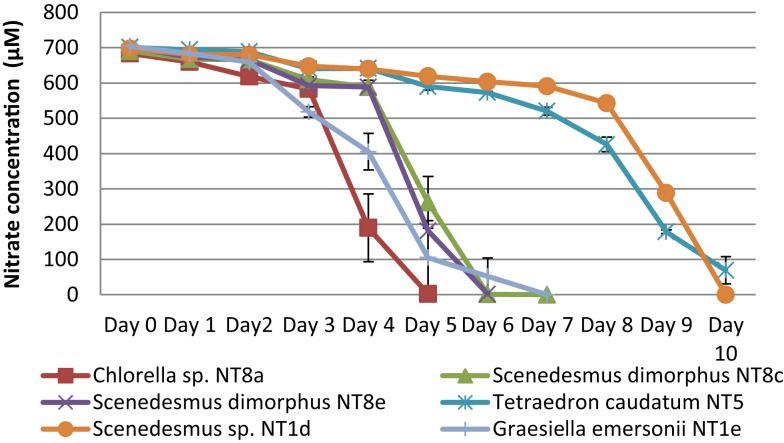
**Nitrogen depletion in cultivation of Northern Territory microalgae experiments**. Shown are mean values and SEs from three separately grown cultures each.

The consumption of nitrogen as a nutrient source for growth is shown in Figure 4. The increase of biomass led to a decrease of nitrogen concentration. This is well illustrated by *Chlorella* sp. NT8a. The log phase of this strain lasted 3 days and was followed by an exponential phase until day 5 after which the cell density was maintained and slightly decreased. Nitrogen intake slightly decreased until day 3 and rapidly decreased below measurable levels after day 5. The growth rates of other strains were lower than *Chlorella* sp. NT8a and the time taken for nutrient depletion was longer.

### Cellular protein contents reached 33%

**Figure 5:**
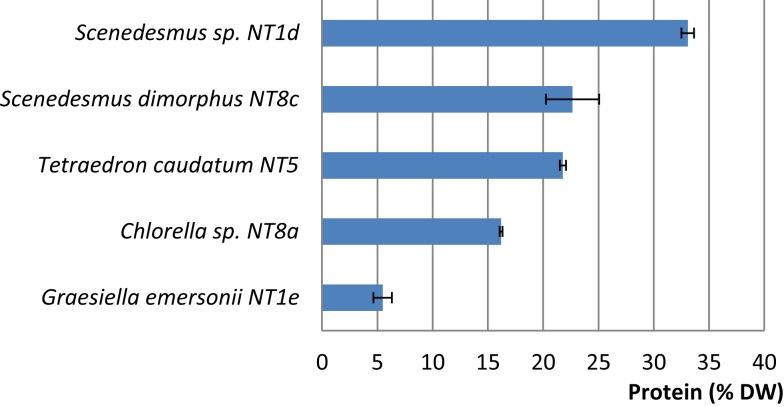
**Protein contents (% DW) in five rapidly growing microalgal strains from the Northern Territory, Australia**. Shown are mean values and SEs from three separately grown cultures each.

Among the five strains assayed, the highest protein content was found in two strains of *Scenedesmus* (*Scenedesmus* sp.: 33.08% DW; *S. dimorphus*: 22.66% DW; Figure 5). *T. caudatum*’s protein content (21.78% DW) was similar to that of *S. dimorphus* (Figure 5).

### Fatty acid profiling and lipid productivity

Gas chromatography/mass spectroscopy (GC/MS) analysis results revealed FAME profiles of the isolated strains (Table 2). *Chlorella* sp. NT8a was the highest FAME producer (116.9 μg mL^−1^ or 14% DW), followed by *S. dimorphus* NT8e (99.13 μg mL^−1^ or 8.2% DW) and *S. dimorphus* NT8c (76.21 μg mL^−1^ or 9.5% DW). The other strains accounted for from 6.08 to 6.95% DW. All analyzed strains produced saturated fatty acids, ranging from 27.05 to 32.58% of the FAMEs; especially C16:0 was the major saturated fatty acid produced by all strains. Unsaturated fatty acids were abundant and ranged from 67.42 to 72.95% of total fatty acids, mostly C18:1, C18:2, and C18:3 (α-linoleic acid – ALA).

**Table 2:** **Fatty acids composition in percentage of total FAME components from Northern Territory microalgae**.

Fatty acids	*Chlorella* sp. NT8a	*Graesiella emersonii* NT1e	*Tetraedron caudatum* NT5	*Scenedesmus* sp. NT1d	*Scenedesmus dimorphus* NT8e	*Scenedesmus dimorphus* NT8c
Lauric (C12:0)	0.30	0.25	0.27	0.26	0.26	0.22
Myristic (C14:0)	0.69	0.09	0.05	1.22	0.23	0.18
Palmitic (C16:0)	33.43	18.79	7.16	9.31	27.94	22.21
Palmitoleic (C16:1)	2.89	2.39	1.43	1.15	2.13	1.90
Hexadecadienoic (C16:2)	2.34	2.40	0.42	0.47	1.15	0.71
Stearic (C18:0)	1.03	2.04	0.46	0.23	1.91	1.59
Oleic (C18:1)	15.09	23.79	6.13	9.24	34.49	24.45
Linoleic (C18:2)	22.29	11.04	3.45	5.48	9.43	6.29
Linolenic (C18:3)	38.85	18.36	11.77	10.38	20.37	17.71
Arachidic (C20:0)	0.00	0.20	0.00	0.00	0.40	0.31
Paullinic (C20:1)	0.00	0.29	0.00	0.00	0.43	0.33
Arachidonic (C20:4)	0.00	0.00	0.00	0.00	0.00	0.00
Eicosapentaenoic (C20:5)	0.00	0.00	0.00	0.00	0.00	0.00
Behenic (C22:0)	0.00	0.23	0.33	0.27	0.39	0.32
Lignoceric (C24:0)	0.00	0.00	1.01	0.00	0.00	0.00
Total FAMEs (μg mL^−1^)	116.90	79.90	32.49	37.99	99.13	76.21
Total FAMEs (% DW)	14.0	6.95	6.5	6.08	8.2	9.5
Saturated fatty acids (%)	30.32	27.05	28.57	29.67	31.4	32.58
Unsaturated fatty acids (%)	69.68	72.95	71.43	70.33	68.6	67.42
Lipid productivity (μg mL^−1^day^−1^)	14.61	9.99	2.71	3.17	12.39	9.53

There was no detection of other valuable unsaturated fatty acids in the isolated strains, such as EPA (eicosapentaenoic acid C20:n5) or DHA (docosahexaenoic acid C22:n6). Lipid productivity among the six strains differed from strain to strain. *Chlorella* sp. NT8a produced the highest amount of triglyceride lipid, 14.61 μg mL^−1^day^−1^, followed by *S. dimorphus* NT8e, 12.39 μg mL^−1^day^−1^, *G. emersonii* NT1e, 9.99 μg mL^−1^day^−1^, and *S. dimorphus* NT8c, 9.53 μg mL^−1^day^−1^. Two other strains, *Scenedesmus* sp. NT1d and *T. caudatum* NT5, displayed the lowest lipid productivity, 3.17 and 2.71 μg mL^−1^day^−1^, respectively.

## Discussion

For a microalgae-based nutrient or oil industry, selection of strains that have high content of protein and lipid is a high priority to achieve commercial production (Lim et al., 2012). Apart from the strain, environmental conditions are the main factors effecting the quantity and quality of microalgal biochemical compounds (Renaud et al., 1994; Salama et al., 2013). In addition, potential strains should be amenable for easy extraction of the biochemical compounds and should be easy to grow in the local environment. Thus, indigenous strains are preferred in terms of expected stable growth, high adaptability for survival and productivity.

The results show that freshwater microalgae that belong to *Chlorophyceae* and *Trebouxiophyceae* were found in almost all Northern Territory samples. The representative species are *S. dimorphus*, *Scenedesmus* sp., *Scenedesmus armatus*, *Chlamydomonas* sp., and *Chlorella* sp. In previous research, Renaud et al. (1994) isolated *Chlorella* sp., *S. dimorphus*, and *Chlamydomonas* sp. in the Alligator River region of the Northern Territory. In present study, besides the strains that Renaud et al. found, we also found some other strains that were abundant in the samples and grew well in BBM medium, such as *T. caudatum* NT5 and *G. emersonii* NT1e. Specific growth rate of *Chlorella* sp. NT8a was higher than previously reported by Lim et al. (2012) and similar to results of Seow et al. (2010).

The protein contents measured in the current study were within the range (11–46% DW) for freshwater algae reported by Boyd (1973). However, higher protein contents compared to the current study were reported in *Chlorella* and *Scenedesmus* strains (Brown, 1991; Becker, 2007; Christaki, 2011). No reports of protein contents in *Tetraedron* and *Grasiella* strains have been published so far. The differences can be due to the variations in culture conditions as factors such as the level of illumination (continuous or 12:12 light:dark cycle), availability of nutrients, growth phase of the algae, etc., can cause different levels of production of proteins (González López et al., 2010; Hempel et al., 2012). The difference can also be due to the differences in protein measurements as the pretreatment methods and subsequent measurement protocol can significantly affect protein contents in different microalgal strains (González López et al., 2010).

All of the selected strains can produce lipids well. For the highest lipid producer, *Chlorella* sp. NT8a, the total fatty acid content and lipid productivity from this study was lower than shown for *Chlorella* by Chisti (2007) (28–32% DW) but higher than previously reported for the same strain by Lim et al. (2012), possibly because BBM medium is considered a better medium for green algae than f/2 medium (Kirrolia et al., 2012). For the FAME components, methyl palmitate (C16:0), methyl oleate (C18:1), methyl linoleate (C18:2), and methyl linolenate (C18:3) were the main components of the total FAMEs analyses. The results are comparable to reported research by Tang et al. (2011) with *Chlorella minutissima*. Of relevance to biodiesel production from the FAME profiling results is the high content of linolenic FAME, reaching an average of 30%. This fatty acid has a high oxidative stability that otherwise may lead to poor stability of fuel (Chisti, 2007). However, fatty acids components can be affected by cultivation factors such as light intensity and nutrients. For instance, C18:3 can be decreased under cultivation conditions of red light and N starvation (Hu et al., 2008; Tang et al., 2011). The lipid profile can be changed based on growth conditions, especially it has been demonstrated numerous times that lipid contents increases if stress conditions of nutrient starvation, UV radiation, and temperature are applied (Boyd, 1973; Lim et al., 2012; Sharma et al., 2012, 2014).

Selection of microalgae for biodiesel production is not only based on growth rate but also on lipid production (together showing the lipid productivity) and suitable fatty acid profiles. *Chlorella* sp. NT8a, *S. dimorphus* NT8c, and *S. dimorphus* NT8e have both high growth rates and lipid contents, especially unsaturated fatty acid content and may present suitable freshwater candidates for biodiesel production. The standard protocol used in the present study also uses a nutrient starvation phase and determined experimentally triglyceride contents and fatty acid profiles at their likely peak. A preliminary understanding of the lipid profile is very useful in selection of potential candidates for larger cultivation. However, the application of these strains for biofuel production requires more research and careful optimization in order to stimulate lipid accumulation. It should be pointed out that this study was designed to isolate, characterize, and directly compare potential strains for the selection of lipid-rich biomass producers under standard (unoptimized) growth conditions. Careful optimization will be required for each strain to improve lipid productivities.

The other point of interest analyzed in this study concerned the ability of Northern Territory microalgae to accumulate high levels of protein. In particular, *Scenedesmus* sp. NT1d, *S. dimorphus* NT8c showed high protein productivities and if amenable to large-scale production could be promising sources for protein-rich animal feed supplement.

## Author Contributions

VD, ST-H, SQ and PS designed the study. VD, SQ and EN acquired and isolated microalgal samples and cultures. VD, FA, ST-H and EN carried out the majority of the experimental work and analysed the data. All authors interpreted the data, contributed intellectually to the presented work and wrote and/or critically revised the manuscript. All authors agree with the final version of the manuscript, including the accuracy and integrity of its contents.

## Conflict of Interest Statement

The authors declare that the research was conducted in the absence of any commercial or financial relationships that could be construed as a potential conflict of interest.
